# Supplementary material for: Comparative genomics provides new insights into the diversity, physiology, and sexuality of the only industrially exploited tremellomycete: Phaffia rhodozyma
Source: BMC Genomics. 2016 Nov 9;17:901. doi: 10.1186/s12864-016-3244-7 (PMC5103461; doi:10.1186/s12864-016-3244-7)
Supplement: Additional file 6: — List of orphan genes with links to PFAM (related to Additional file 1: Table S1). (ZIP 1428 kb) [file 12864_2016_3244_MOESM6_ESM.zip › PFAM_UNIQUE/G03030_P.html]

#                                                                            --- full sequence --- -------------- this domain -------------   hmm coord   ali coord   env coord
  
# target name        accession   tlen query name           accession   qlen   E-value  score  bias   #  of  c-Evalue  i-Evalue  score  bias  from    to  from    to  from    to  acc description of target
  
#------------------- ---------- ----- -------------------- ---------- ----- --------- ------ ----- --- --- --------- --------- ------ ----- ----- ----- ----- ----- ----- ----- ---- ---------------------
  
G03030\_P             -            384 F-box-like           PF12937.2     47    0.0062   14.9   0.0   1   1   9.8e-05     0.016   13.6   0.0    18    46    40    67    11    68 0.91 - F-box-like  
G03030\_P             -            384 GCS2                 PF04107.8    288     0.028   12.2   0.0   1   2     0.092   2.8e+02   -0.9   0.0    93   120    26    52    23    58 0.76 - Glutamate-cysteine ligase family 2(GCS2)  
G03030\_P             -            384 GCS2                 PF04107.8    288     0.028   12.2   0.0   2   2     4e-05      0.12   10.1   0.0    47    84   327   364   298   376 0.90 - Glutamate-cysteine ligase family 2(GCS2)
